# Severe penile torsion of 180 degrees in an adult patient: a uro-radiological case report

**DOI:** 10.25122/jml-2023-0113

**Published:** 2023-10

**Authors:** Abdullah Alzahrani, Abdulaziz Al-Sharydah, Abdulmalik Alkhamis, Mishal Alarifi, Mohammed AlMomen, Abdulaziz Alwarthan, Reem Aldamanhori

**Affiliations:** 1 Department of Urology, College of Medicine, Imam Abdulrahman Bin Faisal University, Dammam, Saudi Arabia; 2 Diagnostic and Interventional Radiology Department, College of Medicine, Imam Abdulrahman Bin Faisal University, Dammam, Saudi Arabia

**Keywords:** penile torsion, urethrogram, magnetic resonance imaging, urethra, penis, stricture

## Abstract

Penile torsion is the abnormal three-dimensional twisting of penile corporal bodies. It can be classified as mild, moderate, or severe, depending on the degree of torsion. Severe penile torsion (>90°) is a very rare condition, with an estimated incidence of 0.4%–1% among all penile torsion cases. Our patient was a 37-year-old man complaining of a 2-year history of lower urinary tract symptoms. These symptoms appeared after the patient sustained an iatrogenic injury during Foley catheter insertion. Physical examination incidentally revealed an obvious counterclockwise penile rotation of 180°. Several theories have been proposed to explain the etiology of penile torsion, including theories based on genetic factors, abnormal urethral development, and abnormal attachment of the dartos fascia to the skin. Penile torsion may be associated with other penile anomalies, including chordee, hypospadias, and epispadias; however, it is often detected as an isolated finding. Clinical examination is sufficient to confirm its diagnosis without the need for further imaging. While no standardized procedure has been indicated for all penile torsion cases, the severity of torsion and the presence of other anomalies determine the most suitable procedure. No reports on the imaging features of penile torsion (irrespective of the degree of torsion) are available. We present the first such report on the imaging features, including advanced magnetic resonance imaging findings, of a 180° penile torsion in an adult patient.

## INTRODUCTION

Penile torsion is the congenital clockwise or counterclockwise twisting of penile corporal bodies. Patients present with varying degrees of rotation; complete reversal of both the corpus cavernosa and spongiosum at the ventral and dorsal penile shafts, respectively, may be observed with or without an aberrant urethral meatus opening [1, 2]. The degree of rotation is measured from the line of the urethral meatus to the scrotal midline and is classified as mild (5°–45°), moderate (45°–90°), and severe (>90°) [3]. Patients with isolated penile torsion, in particular, rarely avail medical care; this is because the condition does not impact their urinary or sexual function. While most cases are discovered during routine examination, cosmetic concerns may encourage patients to actively seek treatment themselves [4, 5]. Clinical examination alone is greatly helpful for diagnosing penile torsion, and imaging studies (such as penile ultrasound or magnetic resonance imaging [MRI] examinations) are generally not performed.

However, radiological examinations could help elucidate the etiology of this anomaly, and a clinical approach for imaging and reporting would prove useful for urologists. Despite the widespread use of ultrasound and cross-sectional imaging in urologic radiology diagnostics, conventional retrograde urethrography still plays a crucial diagnostic role in suspected urethral diseases. It is considered an essential modality for surgical planning and evaluation of postoperative complications. Radiologists must be aware of the anatomical and imaging features of various urethral pathologies as well as of the pitfalls of imaging-based interpretations [6].

The precise and objective measurement of penile curvature or torsion has not been standardized, and an established method for estimating the degree of curvature is unavailable. Established methods should allow real-time estimation of the penile angle for guided intraoperative navigation and decision-making. A standard method for capturing three-dimensional or geometrical digital images using semi-automated algorithms may reliably estimate the penile angle and help urological surgeons with intraoperative decision-making and following up on surgical outcomes [7].

To our knowledge, no study has elucidated the radiological characteristics of penile torsion, especially severe penile torsion (>90°). Herein, we have presented the first imaging characterization (including advanced MRI findings) of a rare case of severe penile torsion of 180°.

## CASE REPORT

**Figure 1:**
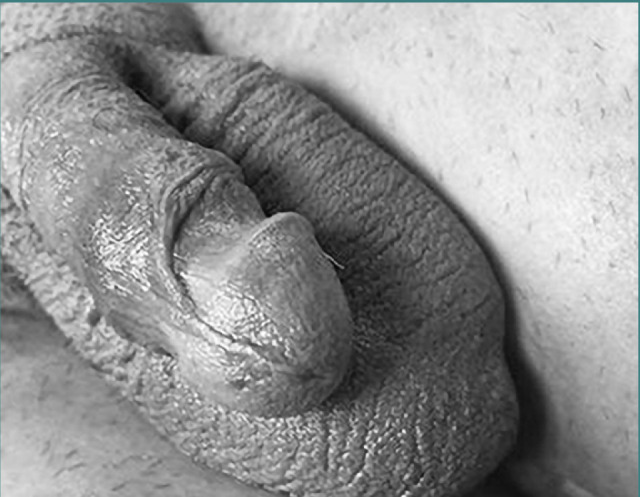
Gross image of the patient’s penis showing a 180˚ counterclockwise torsion

A 37-year-old man, without any prior history of urological conditions or medical interventions, sought medical attention at our clinic due to symptoms consistent with a urethral stricture. The stricture was secondary to an iatrogenic injury sustained during Foley catheter insertion in a left hemicolectomy procedure performed for chronic diverticular disease of the sigmoid colon. The patient, who is married and has two children, denied a history of lower urinary tract symptoms prior to this surgery. Additionally, his sexual history and overall systemic review findings were unremarkable. Genital examination (Figure 1) incidentally revealed a 180° counterclockwise penile rotation with no signs of chordee or hypospadias and no scrotal or testicular abnormalities.

Except for the scar from the previous operation, the chest, cardiovascular, neurological, and abdominal examination findings were unremarkable. All laboratory parameters, including complete blood count findings, were within normal ranges. Urine analysis revealed clear yellow urine with a normal pH and no proteins, red blood cells, glucose, white blood cells, casts, or crystals. Renal function test findings were normal, as follows: blood urea nitrogen,14 mg/dL, serum creatinine, 0.84 mg/dL, and anion gap, 13 mEq/L.

**Figure 2:**
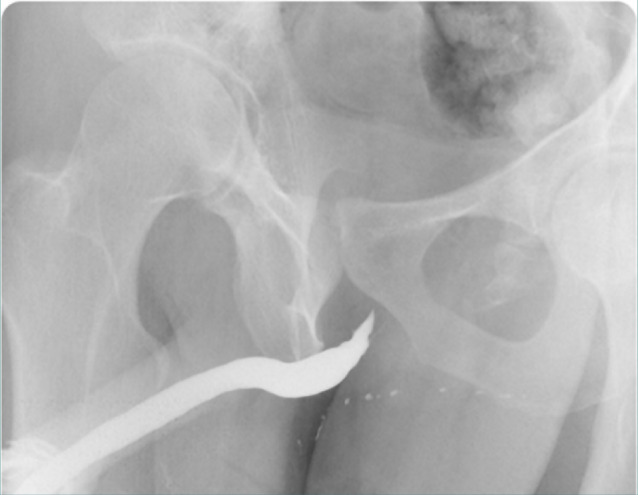
Left anterior oblique projection fluoroscopic static image from retrograde urethrography obtained after injection of a dilute solution of ioptridol (Xenetix 300) contrast in normal saline (1:2) through the urethral opening. The image shows normal opacification of the anterior urethra (bulbar and penile) and an irregular outline with a stricture at the posterior urethra (membranous urethra). No opacification of the prostate urethra is noted. The contrast does not exceed the urogenital diaphragm into the bladder.

A urodynamic analysis revealed the following: maximum flow, 11.5 mL/sec; average flow, 3 mL/sec, voiding time, 2:03 mm:ss.S; flow time 1:23 mm:ss.S; time to maximum flow, 1:29 mm:ss.S; voided volume, 256 mL; pressure at peak flow, 36.6 cmH2O; and flow at peak pressure, 0.3 mL/sec. A retrograde urethrogram revealed a stricture in the proximal urethra (Figure 2).

**Figure 3:**
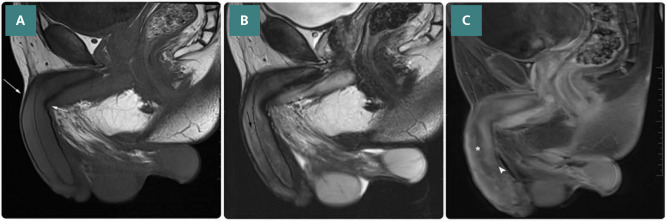
Sagittal MRI scan of the penis. A. T1-weighted spin-echo sequence shows counterclockwisetunical narrowing and torsional twisting at the dorsal and ventral aspects of the proximal penile shaft (white arrows). No fascial discontinuity or uplifting of the superior dorsal vein are noted. B. T2-weighted spin-echo sequence shows heterogenous, multifocal, high-signal intensities distributed across all fascial layers at the dorsal aspect of the corpora (black arrow). C. T1-weighted post-contrast image with fat saturation sequence shows heterogeneous enhancement of the corpora and glans penis, corresponding to areas of chronic tissue ischemia (arrowhead). Additionally, the dorsal-covering fascial layers show intense enhancement secondary to contrast engorgement within the superior dorsal vein. A corresponding lack of central enhancement of the spongy urethra (asterisk) is noted, indicating necrosis as a sequela of chronic ischemia.

**Figure 4:**
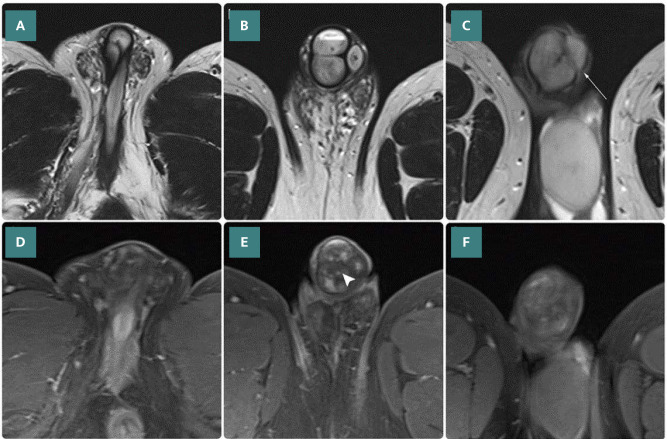
Axial MRI scan of the penis obtained without and with contrast. A, B, and C. T2-weighted sequences obtained at the root (A), body (B), and head (C) levels clearly show counterclockwise torsional twisting of the three erectile structures (corpora), starting from the root (A) and continuing in a 180˚ right-sided torsion. The corpora cavernosa (reversed right to left) and the corpus spongiosum (uplifted) are seen (arrow). D, E, and F. T1-weighted post-contrast image with fat saturation sequence shows heterogeneous enhancement of the corpora and glans penis, corresponding to areas of decreased perfusion and tissue ischemia (arrowheads).

**Figure 5:**
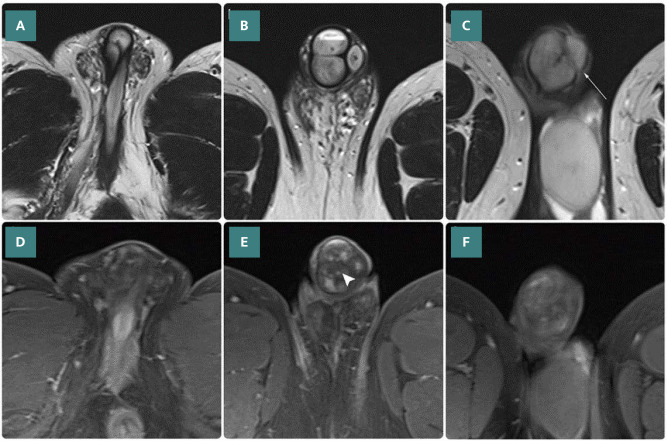
Axial diffusion-weighted MRI scan of the penis. A. Diffusion-weighted sequence, at a high b value of 1,000, shows a small focus of high-signal intensity at the left corpus cavernosum at the body level (arrow). B. Apparent diffusion coefficient map shows the corresponding focus with a lower apparent diffusion coefficient in the same region of interest, reflecting an element of reduced perfusion.

After that, multi-sequential multiplanar dry MRI was performed following gadolinium contrast infusion (Figures 3 A-C, 4 A-F, and 5 A-F).

Comprehensive imaging of the entire penis was performed using MRI, revealing a counterclockwise torsional twisting of the corporal bodies. This torsion originated at the base of the penis and continued in a 180° right-sided torsion. The patient underwent flexible urethroscopy under local anesthesia, which revealed a lesion in the penile urethra with a stricture distal to the bladder neck. The scope easily reached the bladder, indicating that the stricture did not result in any flow obstruction. Consequently, the urethrotome passed through the stricture without difficulty, eliminating the need for urethrostomy, either with or without visual internal urethrotomy.

Despite the patient’s uroflowmetry profile and maximum urinary output, the residual post-void urine volume was insignificant. Furthermore, the patient did not experience any adverse effects related to this rare condition. Therefore, our conservative approach involves regular follow-up appointments at the urology clinic, and behavioral therapy sessions focused on the timing of voiding and double voiding when necessary. However, if the symptoms persist, he should consider undergoing clean intermittent catheterization. After each urethroscopic session, the patient was administered trimethoprim, sulfamethoxazole, nitrofurantoin, and paracetamol.

All treatment options were discussed extensively with the patient despite his reluctance to receive/undergo endogenous agents or procedures. However, a temporary prescription of the spasmolytic agent tamsulosin was sufficient for protecting against possible urine retention [8]. This regimen was continued for 90 days with serial follow-ups at the urology clinic until discontinuation.

## Discussion

We present the first radiological description of a penile torsion identified incidentally during an MRI performed for other reasons. The two corporal cavernosal bodies are observed to lie in the dorsal aspect and appear as intermediate- and high-intensity signals on normal penile T1-weighted and T2-weighted MRI scans, respectively. The corpus spongiosum is found in the ventral area covering the urethra and appears as a low-intensity signal within the higher-intensity corpora.

Penile tissues are enclosed within the following three layers of the fascia: tunica albuginea (covers the cavernosal bodies), Buck’s fascia (surrounds the three penile tissues), and dartos layer (present on the outer surface). The characteristic findings of penile rotation in our case included narrowing in the distal penile area at the level of the tunica albuginea (which was accompanied by twisting of the corporal bodies), while the fascial layers and the superficial dorsal vein ran normally [4].

The incidence of penile torsion is understudied. One study revealed an isolated neonatal penile torsion incidence of 27%; however, the study excluded newborns with torsion of >90° [4]. Conversely, another study revealed an isolated penile torsion incidence of 2% among 5,000 male babies, 1% of whom were diagnosed with severe penile torsion (>90°) [2]. Shaeer found an approximately 12% incidence of penile torsion of varying degrees after studying more than 12,000 adult men; however, none presented with torsion of >90°, and only 2% sought medical attention specifically for the torsion [5].

The theory underlying the etiology of penile malformations remains questionable. Several authors have reported familial cases supporting the role of genetics in this condition [9, 10]. Ahmed *et al*. recently described congenital penile torsion in two siblings, with an isolated 180˚ torsion in the older sibling and an isolated 20˚ curvature in the younger sibling [9].

Bhat *et al*. proposed a relationship between abnormal urethral development and penile torsion, contending that anomalous urethral fold fusion directs the mesodermal covering of the penis with its attachment to one side; this appears as a deviation of the median raphe [11]. Another theory proposes the abnormal attachment of the Dartos fascia to the skin, which may be supported by the absence of penile torsion in cases of proximal hypospadias wherein the ventral skin is absent [10, 12].

Cassell *et al*. recently described a case of a 180° penile torsion associated with distal hypospadias [13]. Although our patient did not have hypospadias, no abnormal alignment of the Dartos fascia was observed. Hence, we support the former theory as the more likely explanation for the aberrant penile rotation.

Although this report represents a novel description of the MRI characteristics of penile torsion, diffusion-weighted MRI revealed areas of penile restriction that are radiologically interpreted as representative of acute ischemic episodes in the penile tissues affected by the torsion (Figure 5).

Therefore, patients with penile torsion should be treated very carefully because this rare condition can affect their quality of life. We recommend a comprehensive approach to follow up with these patients, integrating perspectives from medical, psychosexual, surgical, and reproductive aspects of care [14]. When their growth milestones are reached, these patients should be routinely inquired about concerns regarding their psychological, physical, cultural, social, and sexual levels. Most parameters are modifiable and manageable; however, they cannot be evaluated independently [14].

Surgical options for penile torsion repair have been thoroughly described in the literature. These can be a simple skin incision realignment technique or a more complicated procedure, such as tunica albuginea plication, urethral mobilization, or repair using a Dartos or “V” flap. Nevertheless, no procedure has emerged as superior because the optimal procedure depends on the severity of the torsion and the associated penile or urethral anomalies [1, 3, 5, 11, 15].

## CONCLUSION

Penile torsion is rare and mostly detected during routine clinical examinations; it is seldom associated with other anomalies. Furthermore, severe rotation of >90° is particularly rare. We have presented the first report on the MRI features of a 180˚ penile torsion.
